# Draft Genome Sequence and Annotation of Acinetobacter junii MHI21018, Isolated from Bovine Colostrum

**DOI:** 10.1128/MRA.01700-18

**Published:** 2019-03-07

**Authors:** Carsten Kröger, Kristina Schauer, Seán R. Clerkin, Erwin Märtlbauer, Alastair B. Fleming

**Affiliations:** a Department of Microbiology, School of Genetics and Microbiology, Moyne Institute of Preventive Medicine, Trinity College Dublin, Dublin, Ireland; b Department of Veterinary Science, Faculty of Veterinary Medicine, Ludwig-Maximilians-Universität München, Oberschleiβheim, Germany; University of Southern California

## Abstract

We report here the draft genome sequence of Acinetobacter junii MHI21018, isolated in 2009 from bovine colostrum. The draft genome sequence is composed of 3,267,995 bp, has a GC content of 38.54%, and was assembled into 114 contigs (contig size, >500 bp) with an *N*_50_ value of 72,566 bp.

## ANNOUNCEMENT

Members of the genus Acinetobacter are versatile Gram-negative bacteria. Most notably, multidrug-resistant Acinetobacter baumannii has emerged as a major opportunistic nosocomial pathogen (1, 2). Acinetobacter junii species are rarely associated with human disease, but cases of septicemia caused by A. junii have been reported (3), as well as strains carrying New Delhi metallo-β-lactamase (4, 5) or the carbapenemase-encoding gene *bla*_IMP-1_ (6). To date, 23 A. junii genome sequences are available in GenBank, and only 3 strains are fully sequenced. Due to the rapidly evolving Acinetobacter species, this newly sequenced isolate from an animal source will provide additional information for comparative genomic and evolutionary studies.

Here, we report the draft genome sequence of A. junii MHI21018 isolated in 2009 from the colostrum of a cow which gave birth to a calf that developed bovine neonatal pancytopenia (bleeding calf syndrome). Acinetobacter spp. were selected by colony morphology, a negative oxidase reaction, and by forming red colonies on chromogenic agar (CHROMagar Acinetobacter). The bacteria form round, smooth, opaque, and convex colonies of 2 to 3 mm in diameter when grown on sheep blood agar plates at 37°C for 24 h. After growth of A. junii MHI21018 overnight in 5 ml lysogeny broth (Lennox) at 37°C, genomic DNA was isolated using the Quick-DNA universal kit (Zymo Research). Preparation of the DNA library was carried out using Nextera XT library prep kit (Illumina) following the manufacturer’s protocol. The library was sequenced on the Illumina HiSeq 2500 machine (2 × 250-bp paired-end-reads; total number of reads, 424,136; 30× coverage). Reads were trimmed using Trimmomatic version 0.30 with a sliding window quality cutoff of Q15 (7). Library preparation, sequencing, and read trimming were performed by microbesNG (Birmingham, UK). The draft sequence *de novo* assembly was performed using SPAdes version 3.13.0 in default mode (8), and the properties and quality of the assembly (e.g., *N*_50_, number of contigs, and GC content) were assessed using QUAST version 5.0.2 in default mode (9). Sequences shorter than 200 bp were removed from the final fasta-formatted sequence prior to genome annotation.

The published assembly is composed of 114 contigs (*N*_50_, 72,566 bp), the draft genome size is 3,267,995 bp, and it has a GC content of 38.54%. Species identification was carried out using BLASTn and JSpeciesWS (10). The average nucleotide identity using BLAST (ANIb) returned >97.6% sequence identity with reference A. junii strain 65 (>84% aligned nucleotides); therefore, the sequenced strain was assigned to the A. junii species. Genome annotation was carried out during sequence submission to the NCBI using the Prokaryotic Genome Annotation Pipeline (11), which predicted the presence of 3,341 genes, including 3,086 coding sequences, 68 tRNA genes, 172 pseudogenes, and 1 CRISPR array. Comprehensive Antibiotic Resistance Database (CARD) Resistance Gene Identifier (RGI) analysis predicted the presence of a nitroimidazole antibiotic efflux pump gene (*msbA*) and an ANT(3″)-IIb locus conferring resistance to aminoglycosides (12). PHASTER predicted the presence of one intact prophage (13) with great sequence identity to Psychrobacter phage pOW20-A (NCBI RefSeq accession number NC_020841) and one incomplete prophage, Salmonella phage vB_SosS_Oslo (NCBI RefSeq accession number NC_018279), that are in adjacent chromosomal loci and predicted to partially overlap.

### Data availability.

The raw reads used for the draft genome sequence assembly were deposited in the Sequence Read Archive (SRA) under BioProject number PRJNA509118. This whole-genome shotgun project has been deposited at DDBJ/ENA/GenBank under accession number RWKP00000000. The version described in this paper is version RWKP01000000.
